# Li-Rich Antiperovskite/Nitrile Butadiene Rubber Composite Electrolyte for Sheet-Type Solid-State Lithium Metal Battery

**DOI:** 10.3389/fchem.2021.744417

**Published:** 2021-11-15

**Authors:** Juncao Bian, Huimin Yuan, Muqing Li, Sifan Ling, Bei Deng, Wen Luo, Xuedan Chen, Lihong Yin, Shuai Li, Long Kong, Ruo Zhao, Haibin Lin, Wei Xia, Yusheng Zhao, Zhouguang Lu

**Affiliations:** ^1^ Shenzhen Key Laboratory of Solid State Batteries, Academy for Advanced Interdisciplinary Studies, Southern University of Science and Technology (SUSTech), Shenzhen, China; ^2^ Guangdong Provincial Key Laboratory of Energy Materials for Electric Power, Academy for Advanced Interdisciplinary Studies, SUSTech, Shenzhen, China; ^3^ Guangdong-Hong Kong-Macao Joint Laboratory for Photonic-Thermal-Electrical Energy Materials and Devices, Academy for Advanced Interdisciplinary Studies, SUSTech, Shenzhen, China; ^4^ Department of Materials Science and Engineering, SUSTech, Shenzhen, China; ^5^ Department of Physics, SUSTech, Shenzhen, China

**Keywords:** solid-state batteries, anti-perovskite, solid-state electrolyte, composite electrolyte, sheet-type

## Abstract

Lithium-rich antiperovskites (LiRAPs) hold great promise to be the choice of solid-state electrolytes (SSEs) owing to their high ionic conductivity, low activation energy, and low cost. However, processing sheet-type solid-state Li metal batteries (SSLiB) with LiRAPs remains challenging due to the lack of robust techniques for battery processing. Herein, we propose a scalable slurry-based procedure to prepare a flexible composite electrolyte (CPE), in which LiRAP (e.g., Li_2_OHCl_0.5_Br_0.5_, LOCB) and nitrile butadiene rubber (NBR) serve as an active filler and as a polymer scaffold, respectively. The low-polar solvent helps to stabilize the LiRAP phase during slurry processing. It is found that the addition of LOCB into the NBR polymer enhances the Li ion conductivity for 2.3 times at 60°C and reduces the activation energy (max. 0.07 eV). The as-prepared LOCB/NBR CPE film exhibits an improved critical current of 0.4 mA cm^−2^ and can stably cycle for over 1000 h at 0.04 mA cm^−2^ under 60°C. In the SSLiB with the sheet-type configuration of LiFePO_4_(LFP)||LOCB/NBR CPE||Li, LFP exhibits a capacity of 137 mAh/g under 60 at 0.1°C. This work delivers an effective strategy for fabrication of LiRAP-based CPE film, advancing the LiRAP-family SSEs toward practical applications.

## Introduction

Li ion batteries play critical roles in wide applications of consuming electronic devices, electric vehicles, and large-scale energy storage stations (Scrosati et al., 2011; Cheng et al., 2017; Manthiram, 2017). Solid state batteries have been believed to be one of the most important next-generation battery technologies due to their large energy density and outstanding safety (Janek and Zeier, 2016; Manthiram et al., 2017; Xin et al., 2017; Xia et al., 2019). Using solid electrolyte to replace the flammable liquid electrolyte can largely relieve the thermal runaway risk of the conventional Li ion batteries (Janek and Zeier, 2016; Xia et al., 2019; Chen et al., 2020a; Gao et al., 2020). Currently, solid-state electrolytes (SSEs) include argyrodite (e.g., Li_6_PS_5_Cl) (Deiseroth et al., 2008), sulfides (e.g., Li_9.54_Si_1.74_P_1.44_S_11.7_Cl_0.3_) (Kato et al., 2016; Xiao et al., 2021), LISICON (e.g., Li_2+x_Zn_1-x_GeO_4_) (Adachi et al., 1996), NASICON (e.g., Li_1.3_Al_0.3_Ti_1.7_(PO_4_)_3_) (Deng et al., 2015), garnet (e.g., Li_7_La_3_Z_r2_O_12_) (Murugan et al., 2007; Huang et al., 2020; Huo et al., 2020), perovskite (e.g., Li_0.5_La_0.5_TiO_3_) (Conductor et al., 2000), and Li rich anti-perovskite (LiRAP, e.g., Li_3_OCl_0.5_Br_0.5_) (Zhao and Daemen, 2012; Emly et al., 2013; Li et al., 2016a; Hood et al., 2016; Zhu et al., 2016; Xu et al., 2019; Yin et al., 2020) have been widely investigated. Their Li ion conductivities are in the levels of 10^–6^ ∼ 10^–2^ S cm^−1^ with the activation energy being in the range of 0.2–0.6 eV. Considering the Li ion conductivity of the commercial liquid electrolyte (∼10^–2^ S cm^−2^), only the sulfide-based SSEs can reach that level. For example, Li_9.54_Si_1.74_P_1.44_S_11.7_Cl_0.3_ (Kato et al., 2016) and Li_7_P_3_S_11_ (Seino et al., 2014) have the ionic conductivities of 2.5 × 10^–2^ S cm^−2^ and 1.7 × 10^–2^ S cm^−2^, respectively. However, the sulfide-based SSEs have poor electrochemical stability, poor air stability, and are facing great challenges in large-scale production and commercialization (Janek and Zeier, 2016; Chen et al., 2020b; Zhao et al., 2020).

As an alternative, LiRAP superionic conductors, e.g., Li_3_OCl and Li_3_OCl_0.5_Br_0.5_, represent a new family of SSEs. It contains Li^+^ up to 60 at.%. The Li ion conductivity can be up to 1.94 × 10^−3^ S cm^−1^ with the activation energy of 0.2–0.3 eV (Zhao and Daemen, 2012). More favorably, synthesis of LiRAPs can be easily realized by mixing LiCl, Li_2_O (or LiOH) and LiBr and heating the mixtures up to 300–600°C (Zhao and Daemen, 2012; Emly et al., 2013; Li et al., 2016b; Hood et al., 2016), which makes LiRAPs very cost-effective and the production be easily scaled up. These properties ensure LiRAPs competitive candidates for the applicable solid electrolytes. Currently, all-powder-based SSLiBs face a great challenge to form two-dimensional contacts between the active electrode materials and the SSEs (Han et al., 2018; Shao et al., 2018; Feng et al., 2019). A sintering procedure is needed to reduce the grain boundary induced resistance and enhance the wettability of the SSE with electrode materials (Lu et al., 2018; Duan et al., 2019). Moreover, the contact losses among the battery components due to the volume changes of active materials upon cycling is one of the crucial failure mechanisms (Yao et al., 2019; Zhang et al., 2020a; Popovic et al., 2021). Recently, SSE/polymer composites, namely composite electrolytes (CPEs), have emerged as attractive techniques as they combine the advantages of inorganic SSEs (high ionic conductivity and mechanical strength) and solid polymer electrolyte (SPE, good interfacial properties and flexibility) (Chai et al., 2018; Oh et al., 2019; Yao et al., 2019; Zhang et al., 2020a; Zhang et al., 2020b; Fan et al., 2020; Chen et al., 2021; Popovic et al., 2021). Preparation of CPE film relates to slurry-based methodology, which can be easily scaled up. This technique is desirable for advancing the practical applications of LiRAP SSEs, but is still yet to be explored.

To fill this technique gap, a slurry-based procedure for preparation of CPE with LiRAP SSEs, e.g., Li_2_OHCl_0.5_Br_0.5_ (LOCB) as an active filler and nitrile butadiene rubber (NBR) as a polymer scaffold, is developed in this work. Addition of LOCB into the polymer electrolyte enhances the Li ion conductivity and reduces the activation energy. The as-prepared LiRAP/NBR CPE film shows good chemical stability, thermal stability, flexibility, and mechanical property. It is electrochemically stable in the range of 0∼5 V vs. Li/Li^+^. The Li symmetric cell with the CPE as separator can work for more than 1000 cycles with minor polar voltage changes. This work provides a new approach to fabricate LiRAP based sheet-type SSLiBs and advances the LiRAP SSEs toward practical applications.

## Experimental

### Preparation of LOCB Powder

All the chemicals were purchased from Aladdin without additional statement. The LOCB SSE was prepared as referred to in the previous report (Zhao and Daemen, 2012; Hood et al., 2016). Briefly speaking, LOCB was synthesized by solid-state reaction of the mixture of LiCl (99.9%), LiBr (99.99%), and LiOH (99.9%). The molar ratio of LiCl, LiBr, and LiOH is 1:1:2.4. The mixture was carefully ground by hand for 0.5 h. Then it was pelletized and put into a Ni crucible. They were placed into a furnace positioned inside an Ar filled glovebox and heated at 500°C for 10 h. Next, the product was cooled with the furnace to room temperature (RT). The product was hand-milled at first and then ball-milled with the addition of dibromomethane (DBM) at 400 rpm/min for 5 h.

### Preparation of LOCB/NBR CPE Film

NBR (M.W, ∼3800, acrylonitrile 37–39 weight ratio, wt.%, Sigma Aldrich) was dried under vacuum at 80°C for 12 h and then stored in the glove box. In a typical preparation, 0.5 g of NBR bulks was added into 9.5 g of DBM. Then the mixture was stirred at 1000 rpm and 60°C for overnight. Finally, a viscous NBR gel was obtained. Equal weight of LOCB and LiTFSI [3M in triethylene glycol dimethyl ether (TEGDME, 99%)] was added into the NBR gel and stirred at 2000 rpm for 10 min by planetary centrifugal paste mixer (Thinky SR-500). The wt.% of LOCB in all solid components is tuned from 0 to 80% with an interval of 20%. DBM can be added to tune the viscosity of the slurry to facilitate the coating. The slurry was coated on silicone paper by the doctor-blade method and dried at 60°C for 6 h. Except for the mixing procedure on the planetary centrifugal paste mixer, all the procedures were conducted in the Ar filled glove box (<0.1 ppm O_2_, <0.1 ppm H_2_O). The chemical stabilities of different organic solvents including ethanol (99%), N-methyl pyrrolidone (NMP, 99%), DBM (99%), acetonitrile (AN, 99%), and tetrahydrofuran (THF, 99%) with LOCB were also investigated.

### Materials Characterization

Surface/cross-sectional morphologies of the samples were measured on a scanning electron microscope (SEM, FEI Nova NanoSEM 50). X-Ray Diffraction (XRD) patterns were recorded on a Smartlab X-ray diffractometer (Cu K_α_, *λ* = 1.5406 Å, Rigaku, Japan). The samples were placed onto the silicon sample holder and sealed by polyimide film. Thermogravimetric (TG) curves were measured on a TG analyzer (NETZSCH, STA449F3 Jupiter). Compositions of the samples were investigated by FTIR spectroscopy (Perkin Elmer).

### Preparation of the SSLiB With Sheet-Type Electrode

Wet slurry consisted of LiFePO_4_ (LFP, 78 wt%), super P (5 wt%), carbon nanotube (3–5 μm in length, 5 wt%), NBR (10 wt%), lithium bis(trifluoromethane)sulfon imide (LiTFSI, 2 wt%), and DBM was coated on Al foil and dried at 60°C for 6 h. The LFP electrode (areal capacity: 0.9 mAh cm^−2^) and the Li foil were placed on each side of the CPE film. The coin-type SSLiBs were sealed at 50 MPa at RT. For the pouch cell assembling, Ni and Al conductors were welded with Cu and Al foils, respectively, by ultrasonic spot welder. Cu foil was attached to the surface of Li foil at the other side. The sealing procedure was carried out on a vacuum sealing machine. Except for the slurry mixing procedure on the planetary centrifugal paste mixer, all the procedures were conducted in the Ar filled glove box (<0.1 ppm O_2_, <0.1 ppm H_2_O).

### Electrochemical Characterization

Stainless steel (SS)||CPE film||SS symmetric cells were assembled for the EIS test. The EIS plots were measured under an amplitude voltage of 20 mV and the frequency range from 0.1 Hz to 1 MHz using a Solartron electrochemical workstation (1470E). The ionic conductivity (*σ*) is calculated by the equation:

$$\sigma =\frac{d}{AR}$$

where *d* is the thickness of the film, *A* is the area, and *R* is the bulk resistance of the film. Pt||CPE film||Li cells were assembled for the Cyclic Voltammetry (CV) test. The CV scans started from 2 toward 5 V and scanned in the range of 0–5 V vs. Li/Li^+^ for two cycles. Galvanostatic charge–discharge cycling of the Li||CPE||Li cells was performed at 60°C on the NEWARE battery test system. The diameter of the Li foil is 8 mm. The thickness of the CPE film is 310 μm. Galvanostatic charge–discharge cycling of the SSLiBs was conducted at 60°C between 2.8 and 3.8 V (vs. Li/Li^+^) on the LAND battery test system. Direct current polarization curves were measured at 0.5 V for 1,800 s to measure electric conductivity of CPE film in the SS||CPE||SS cells.

## Results and Discussion

**FIGURE 1:**
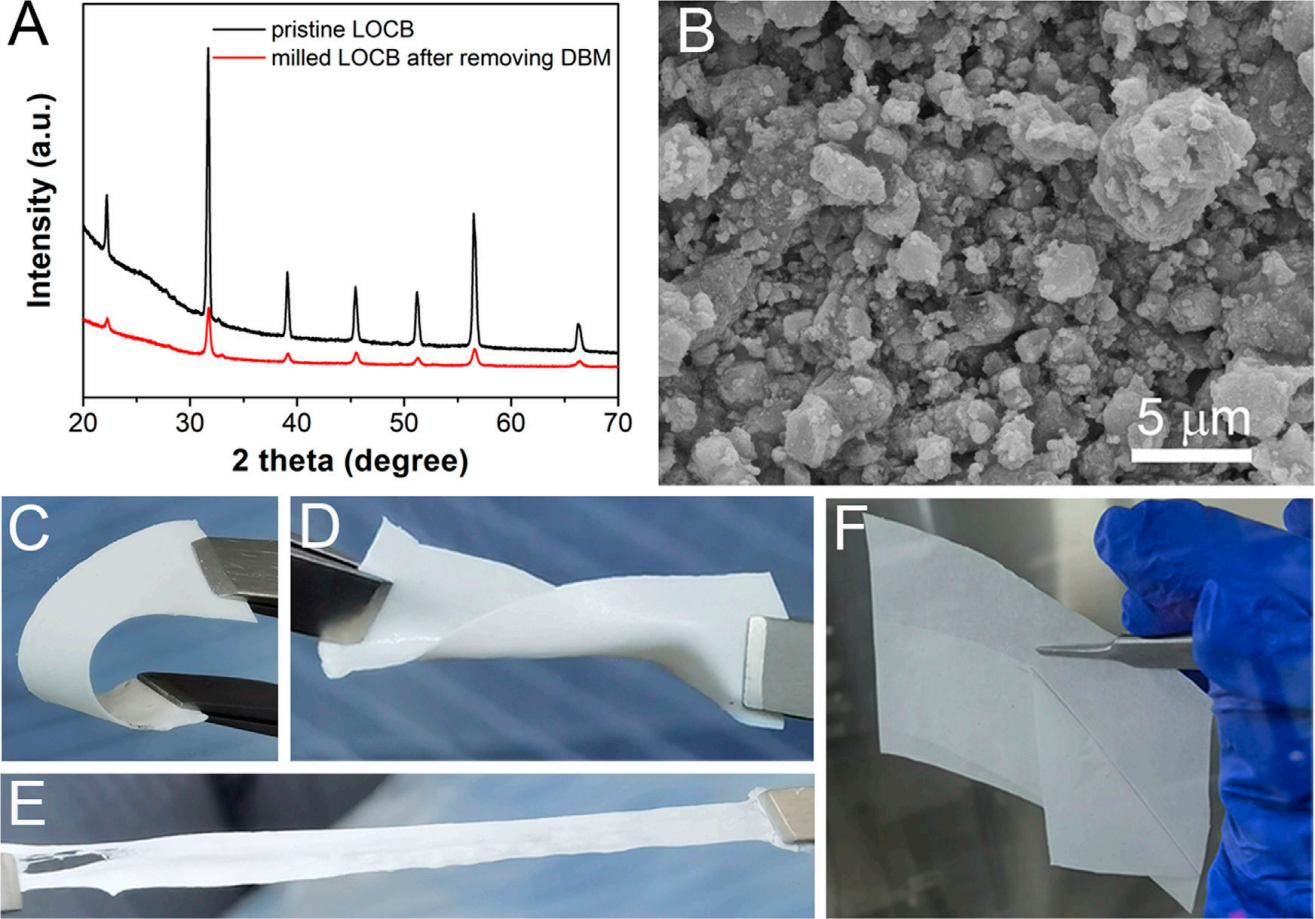
**(A)** XRD patterns of LOCB, **(B)** SEM image of the milled LOCB powders. **(C–E)** Bending, distorting, and stretching of the LOCB/NBR CPE films. **(F)** Detaching of the CPE film from silicone paper.

To develop a slurry-based procedure to fabricate LiRAP based composite electrolyte, the organic solvent should be carefully chosen to avoid the side reactions. It is well known that when exposing it in the air, LiRAP will rapidly decompose, producing Li_2_CO_3_ and lithium halide (Hanghofer et al., 2018). However, the stability of LiRAP with organic solvent is rarely investigated. Strong Lewis-basic or highly polar solvents with highly electronegative elements (e.g., as O and N) have lone-pair electrons, which may easily react with the electrophilic species of LiRAP (e.g., -OH) (Oh et al., 2019). To test this, we mixed LOCB powder with some frequently used organic solvents including ethanol, NMP, DBM, AN, and THF. After storing the mixtures in the Ar filled glove box for 7 days at RT, obvious color change of AN and THF are observed (Supplementary Figure S1), indicating their side reactions with LOCB. XRD patterns of the LOCB powders dried from ethanol, NMP, AN, and THF reveal a lot of new peaks originated from the reaction products (Supplementary Figure S2). Only DBM is stable with LOCB as the diffraction peaks of LOCB are identical before and after mixing with DBM, as shown in Figure 1A. By referring the polarity of these solvents, it is found that DBM has the lowest polarity (*p* = 3.8, Supplementary Table S1). Therefore, we infer that the polarity of the organic solvent should be less than 4 to ensure the compatibility of the solvent with LiRAP. To confirm this, we mixed p-xylene (*p* = 2.5) with LOCB powder for 7 days. No additional diffraction peak is found (Supplementary Figure S3), demonstrating that polarity is a key factor for the choice of the organic solvent. In this work, DBM is chosen as the solvent as it is miscible with TEGDME based electrolyte (Supplementary Figure S4).

Before coating the CPE film, it is desirable to decrease the size of the solid electrolyte, which is beneficial for the uniformity and Li ion conductivity of the CPE film (Zhang et al., 2016). DBM is added when milling the LOCB powders to prevent the agglomeration of the LOCB particles (Supplementary Figure S5). It is found from the SEM image (Figure 1B) that the size of the milled LOCB particles ranges from tens of nanometers to several microns. NBR is selected as the polymer scaffold with the following considerations. First, it can be easily dissolved in low polar solvent (Nam et al., 2018; Oh et al., 2019). Second, it contains nitrile groups, which enables its high dielectric constant, high anodic oxidization potential, and favorable interaction with Li^+^ (Hu et al., 2016). Moreover, it has good viscidity, which enables it a strong scaffold when mixing it with inorganic materials. The transparent NBR film (Supplementary Figure S6) turns to be white (Figures 1C–F) after the addition of white LOCB particles. The LOCB/NBR CPE films exhibit good flexibility. It can be bended, distorted, and stretched, as presented in Figures 1C–E. Moreover, the as-prepared films are detachable from silicon paper (Figure 1F). These properties ensure that the LOCB based CPE films can be utilized in sheet-type pouch SSLiBs. The TG curves (Supplementary Figure S7) confirm the improved thermal stability of the LOCB/NBR based CPE film compared with the pure NBR film.

**FIGURE 2:**
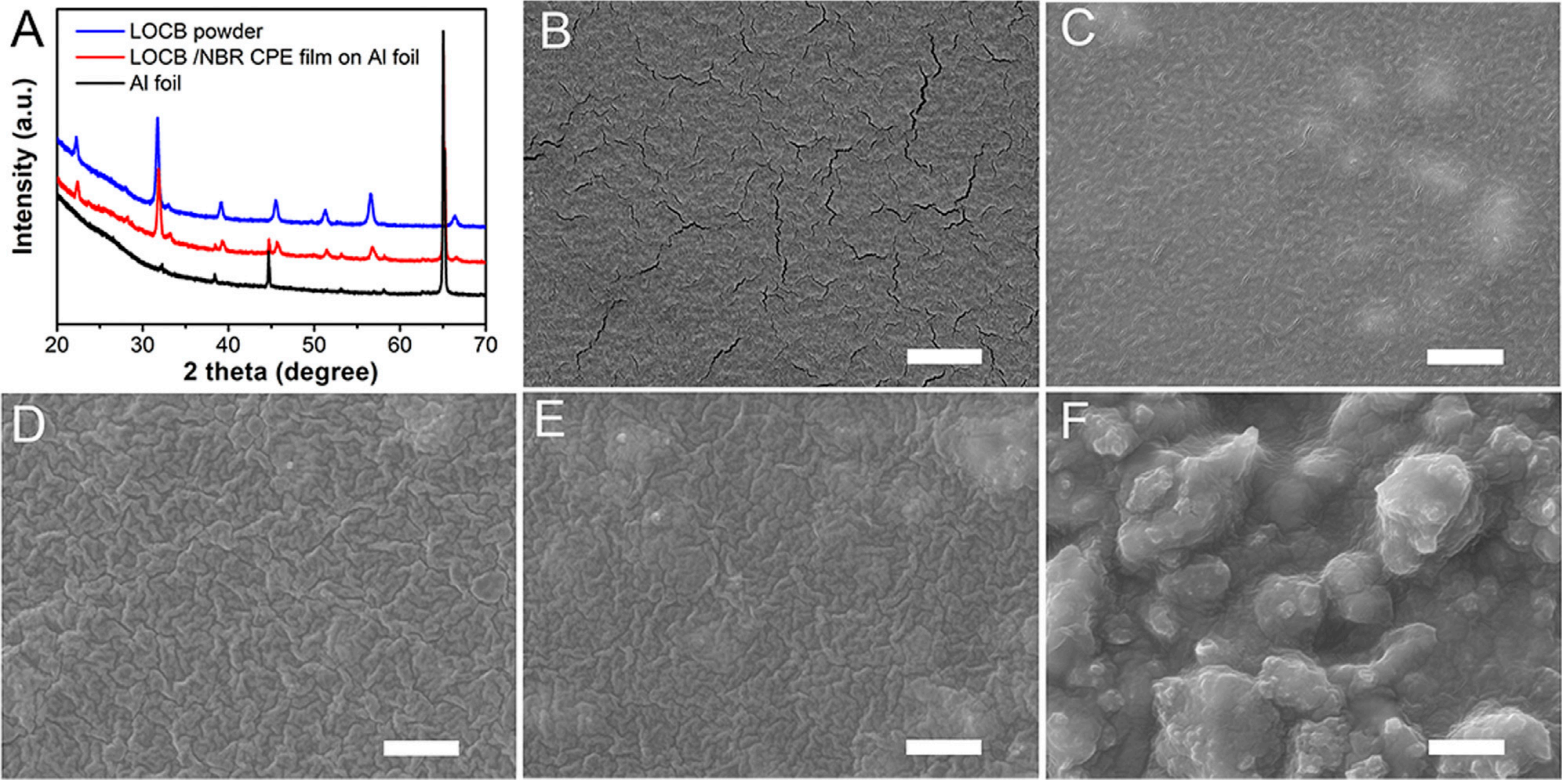
**(A)** XRD pattern of the pure Li_2_OHCl_0.5_Br_0.5_, Li_2_OHCl_0.5_Br_0.5_/NBR CPE film, and Al foil. **(B–F)** SEM images of the NBR SPE and Li_2_OHCl_0.5_Br_0.5_/NBR CPE films with different LiRAP ratios. **(B)** 0, **(C)** 20, **(D)** 40, **(E)** 60, and **(F)** 80%. The scale bars are 2 µm.

The XRD pattern of the LOCB/NBR CPE film, as illustrated in Figure 2A, is identical to that of the pristine LOCB powder. It demonstrates no chemical change of the LOCB during slurry preparation and film coating procedures, indicating good chemical compatibility of LOCB with DBM, liquid electrolyte, and NBR. Differences in the surface morphology of the CPE film can be observed when tuning the ratio of the LOCB, as shown in Figures 2B–F. For NBR SPE film without the addition of LOCB, there are a lot of cracks on the surface, as is shown in Figure 2B, which is attributed to the shrinkage stress during the drying procedure. With an increase in the LOCB ratio, the number of the cracks gradually decreases. The cracks disappear when the ratio is 40% (Figure 2D). This can be ascribed to the incorporation of LOCB particles, which enhances the mechanical strength of the composite. One can also find in Figures 2C–E that there are some blurry islands on the surface, which originates from the LOCB microparticles. Further increasing the content of LOCB to 80wt.% results in a very rough surface. It means that such kind of film cannot contact intimately with the electrode layers, which will bring about large interface resistance.

**FIGURE 3:**
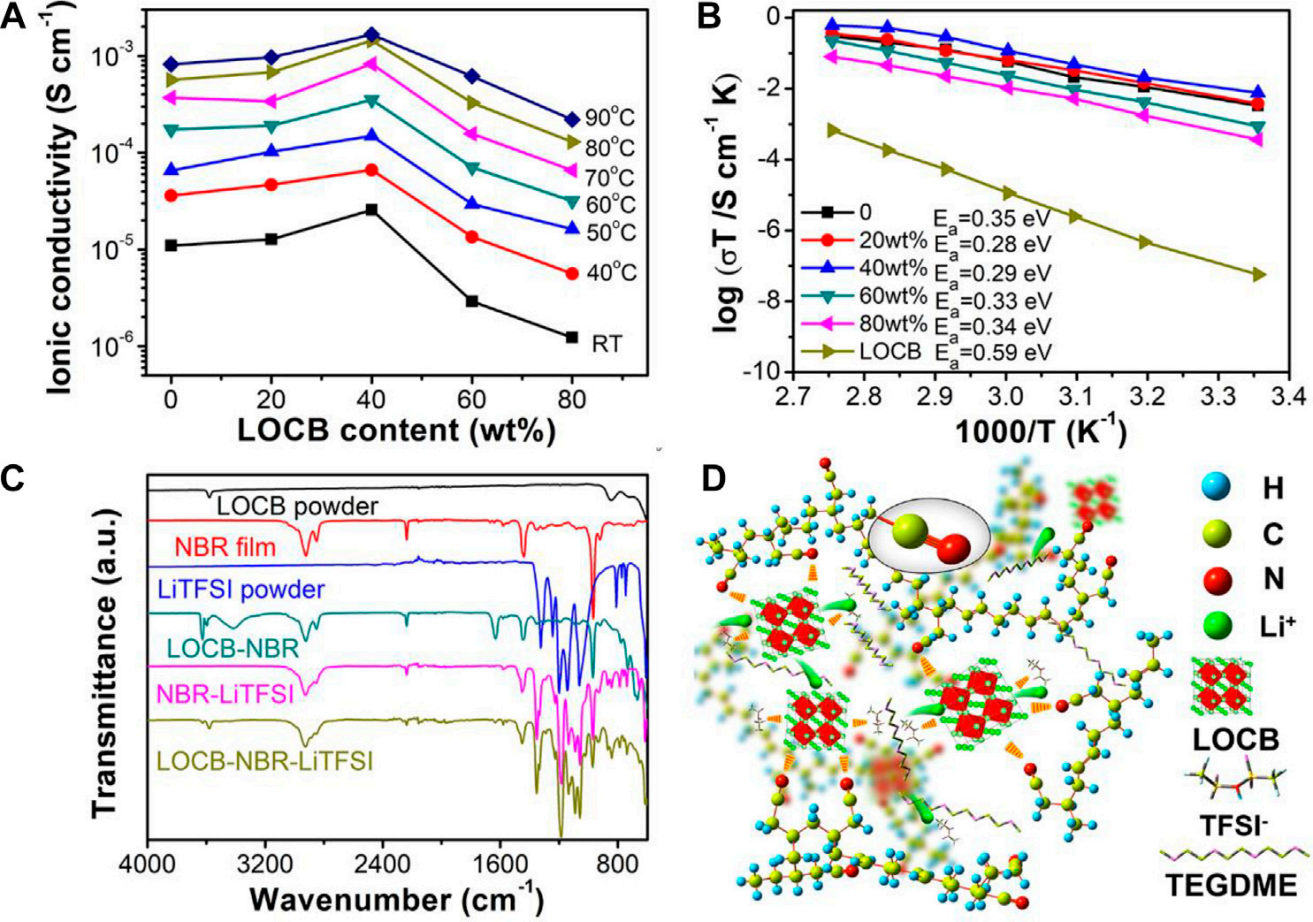
**(A)** LOCB content ratio dependent ionic conductivity of the CPE films at different temperatures. **(B)** Arrhenius plots of CPE films with different LOCB content ratio. **(C)** FTIR spectra of the different components of LOCB/NBR CPE film and their mixtures. **(D)** Schematic illustration of the Li ion conduction mechanism of the LOCB/NBR CPE film.

The ionic conductivity of the LOCB powder is cal. 2.6 × 10^–6^ S cm^−1^ at RT. Figure 3A shows the LOCB wt.% (from 0 to 80wt.%) dependent ionic conductivities of the LOCB-NBR CPE films measured from RT to 90°C. With an increase of the LOCB content, the CPE film undergoes a “ceramic-in-polymer” to “polymer-in-ceramic” change. The ionic conductivity of the CPE film initially increases and reaches the maximum at the LOCB content of 40wt.% in all tested temperatures ranging from RT to 90°C, which is consistent with the reported CPE films with other SEs (Zhang et al., 2017; Chen et al., 2018). At RT, the ionic conductivity of the NBR SPE film is 1.1 × 10^–5^ S cm^−1^, while it is 2.6 × 10^–5^ S cm^−1^ for 40wt.% LOCB/NBR CPE film. The ionic conductivity of 40wt.% LOCB/NBR CPE film reaches 1.4 × 10^–3^ S cm^−1^ at 80°C. Further increasing the LOCB content to 60 and 80wt.% reduces the ionic conductivity, even less than that of the NBR SPE. This phenomenon is due to the destruction of the NBR ion transport paths. In such a case, LOCB particles turn to the dominant components of the CPE, which will aggregate in the film. The ion conduction among the LOCB particles is much slower than in the bulk of LOCB. When the LOBC content is 80wt.%, the surface of the CPE becomes very rough (Figure 2F). Hence, the film cannot intimately contact with the steel, which further reduces the ion conductivity.

Figure 3B presents the Arrhenius plots of the CPE films with different LOCB contents. The activation energy (E_a_) was calculated according to the Arrhenius equation *σ*T = Aexp(−E_a_/kT) (Zhao and Daemen, 2012), where k is the Boltzmann constant, *σ* is the ionic conductivity of SSEs, and T is the absolute temperature. The E_a_ of NBR SPE is 0.35 eV. The addition of LOCB obviously decreases E_a_ (0.28 eV for 20wt.% LOCB and 0.29 eV for 40wt.% LOCB), which is ascribed to the interaction between LOCB and NBR. Further increasing the LOCB contents, E_a_ gradually increases due to the larger E_a_ of LOCB (0.59 eV).

To further investigate the ionic conduction mechanism of the LOCB/NBR CPE film, FTIR spectra are measured to explore the interactions of the different components of the CPE film. As is shown in Figure 3C, for NBR film, the vibration peak at 2236 cm^−1^ is assigned to the stretching mode of -C≡N groups. A weak peak at 2262 cm^−1^ appears in LOCB-NBR, NBR-LiTFSI, and LOCB-NBR-LiTFSI compared with pure NBR film (Supplementary Figure S8), which originates from the interactions between -C≡N group of NBR and Li^+^ from LOCB and LiTFSI (Verdier et al., 2020). Moreover, the vibration peaks of LiTFSI in the range of 1400∼500 cm^−1^ shows red or blue shift when compared with those of NBR-LiTFSI (Supplementary Figure S9). However, the characteristic peaks of LiTFSI in the FTIR spectra of NBR-LiTFSI and LOCB-NBR-LiTFSI are identical, indicating that TFSI^−^ groups only interact with NBR. The vibration peak of LOCB powder at 3576 cm^−1^ is assigned to -OH (Li et al., 2016b). This peak disappears while new peaks at 3628, 3600, 3413, 1632, 730, 665, and 594 cm^−1^ appear, indicating strong interactions between LOCB and NBR. The above interactions largely facilitate the segmental motion of NBR chains, which enhances the Li^+^ conductivity of the NBR SPE film. Moreover, without the addition of Li salt electrolyte, the NBR/LOBC mixture film exhibits ionic conductivity of less than 10^−9^ S cm^−1^ (Supplementary Figure S10). As confirmed by the TG curve shown in Supplementary Figure S7, there is residue TEGDME inside the CPE films. The dissolved LiTFSI in the TEGMDE forms important paths for Li^+^ transport.

According to the above analysis, a possible CPE microstructure and Li transport pathways are proposed, as illustrated in Figure 3D. Under the effect of an electric field, the Li^+^ tends to hop from one coordinating site to another. This hopping is facilitated by the dissolved LiTFSI in the TEGMDE, segmental motions of nitrile groups in the NBR chains or an C≡N-Li^+^ cluster-assisting function. The segmental motion of the NBR chains offers free spaces for Li^+^ migration (Chen et al., 2016; Zhao et al., 2018). After the addition of LOCB into the NBR SPE, the -OH groups in LOCB will destroy the balance of -C≡N-Li^+^ clusters. They will attract the C≡N- and TFSI^−^ groups, resulting in the decreased attracting force of C≡N- and TFSI^−^ groups to Li^+^. Therefore, after the addition of LOCB, the E_a_ values decrease. We speculate that LOCB can affect the motion of the NBR chains due to the existence of the polar -OH groups, which may provide more space for Li^+^ transport.

**FIGURE 4:**
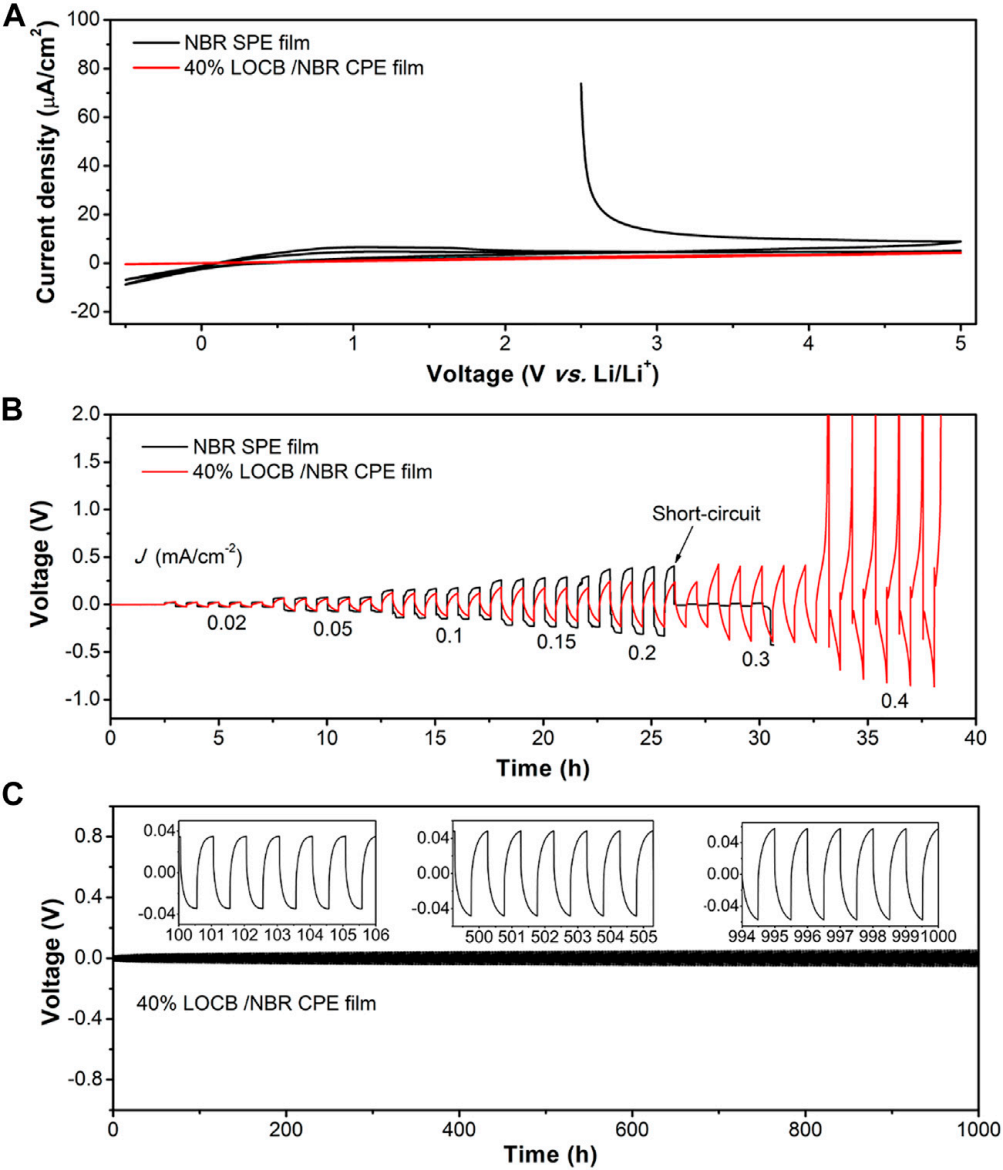
Electrochemical properties of NBR SPE film and LOCB/NBR CPE film. **(A)** Cyclic voltammetry curves and **(B)** galvanostatic cycling of Li-Li cells at different current densities. **(C)** Galvanostatic cycling of Li-Li cell at 0.04 mA cm^−2^. The insets in **C** present the detailed polar voltage at specific cycles.

Taken the ionic conductivity, mechanical strength, and the surface roughness into account, we chose the CPE film with 40wt.% LOBC for further investigation. To investigate the electrochemical stability of the NBR SPE film and LOCB/NBR CPE film, CV curves were conducted, as shown in Figure 4A. It is found that the reduction and oxidation current of NBR SPE film are −8.75 μA cm^−2^ at 5 V vs Li/Li^+^ and 8.9 μA cm^−2^ at 0 V vs. Li/Li^+^, much larger than that of the LOCB/NBR CPE film (0.4 μA cm^−2^ at 0 V vs. Li/Li^+^ and 4.2 μA cm^−2^ at 5 V vs. Li/Li^+^), demonstrate an improved electrochemical stability of the CPE film. The improved stability is because LOCB particles enhance the dielectric property of the NBR film, which block the electron transport and suppress the oxidation/reduction reactions. The calculated electric conductivity of the NBR SPE film and the CPE film are 1.7 × 10^–9^ and 2.1 × 10^–10^ S cm^−1^, respectively (Supplementary Figure S11). In the second scan cycle, the currents decrease as the surface reaction products passivate the surface, relieving the surface reactions.

The Li dendrite endurance ability of the CPE film was evaluated by galvanostatic cycling of the Li-Li symmetric cells at step-increased current densities at 60°C. Figure 4B presents the voltage-time profiles of the 40wt.% LOCB/NBR CPE film and the NBR SPE film. The voltages increase with the currents for both due to the polarity. When the current density reaches 0.2 mA cm^−2^, a voltage drop is observed for NBR SPE film, which results from the penetration of the Li dendrites across the film. It is because the Li dendrites can penetrate the film very easily through the cracks, as is shown in Figure 2B. In contrast, the voltage drop does not appear even at 0.4 mA cm^−2^ for the CPE film. Instead, the polar voltage surpasses 5 V at 0.5 mA cm^−2^, indicating an elevated critical current density. This phenomenon demonstrates that the CPE film has a strong ability for preventing the Li dendrite penetration. The interface stability of 40wt.% LOCB/NBR CPE film with Li metal anode was further assessed by galvanostatic cycling procedure in Li-Li symmetric cell, which was charged and discharged at the current density of 0.04 mA cm^−2^ for 0.5 h, respectively, at 60°C. The voltage change vs. time is presented in Figure 4C. After 1000 cycles, the voltage change is only 0.03 V, indicating a relatively stable interface between the CPE film and Li anode.

**FIGURE 5:**
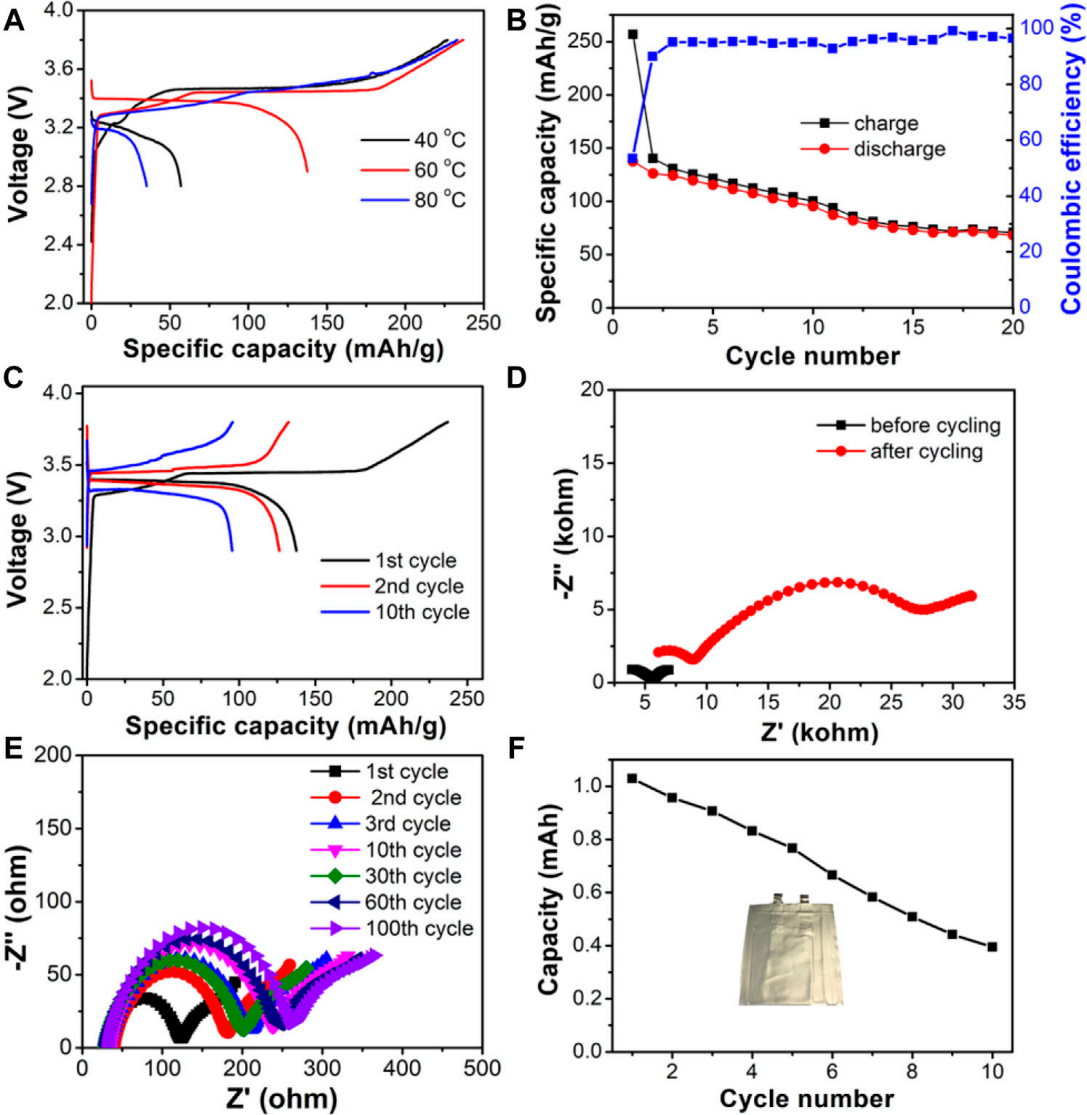
**(A–E)** Electrochemical performance of Li/CPE/LiFePO_4_ coin cells based on the composite electrolyte. **(A)** Voltage-capacity curve of the first cycle at different temperatures. **(B)** Cycling performance at 60°C. **(C)** Voltage-capacity curve of different cycles at 60°C. **(D)** EIS plots of the SSLiB before and after cycling measured at 60°C. **(E)** EIS plots of the Li-Li symmetric cell running for different cycles. **(F)** Pouch cell performance.

The formation processes of the batteries under different temperatures were investigated to optimize the running conditions of the LOCB/NBR CPE film based SSLiB. The discharged capacity at the first cycle augments when the temperature increases from 40 to 60°C, as shown in Figure 5A. Further elevating the temperature to 80°C reduces the discharge capacity. The first cycle discharge capacities at 40, 60, and 80°C are 57, 137, and 35 mAh/g, respectively, with the corresponding coulombic efficiencies (CEs) of 25.2, 53.3, and 15.0%. The low CEs are ascribed to the oxidation/reduction reactions at the electrode surface, as is detected in the CV curves shown in Figure 4A. Herein, the enhanced discharge capacity from 40 to 60°C is mainly attributed to the improved Li ion conductivity, as shown in Figure 3A. When the temperature is further increased to 80°C, although the Li ion conductivity is raised, the side reactions at the electrode material/CPE interface are also intensified, which lead to the degraded capacity. According to the above results, 60°C is chosen as the running temperature. Figure 5B presents that after cycling for 20 times, the discharged capacity retains 49.5% of that at the first cycle. The voltage-capacity curves of different cycles depicted in Figure 5C show that the polar voltage constantly increases with the charge/discharge process. EIS plots, as are shown in Figure 5D, deliver that the impedance, especially the interface resistance (the semicircle at low frequency), largely increases, which leads to the degraded capacity. After disassembling the battery, it is found that the intimate contact between Li and CPE is still maintained (Supplmentary Figure S12). However, the cathode becomes more loosened (Supplmentary Figure S13), which is possibly due to the destruction of the sticky group of NBR from the cathode oxidation. The EIS plots of the Li/CPE/Li symmetric cells after running for different cycles show that the Li-CPE interface impedance (corresponding to the semicircle at low frequency) becomes stable after three cycles, as is shown in Figure 5E and Supplmentary Figure S14. It demonstrates that the reaction products of Li-CPE film can serve as stable solid electrolyte interphase (SEI) layer and block the further reaction of CPE film with Li foil. The slight increase of the bulk resistance at high frequency is due to the sluggish reaction of LiRAP particles with the TEGDME molecules, which decreases the ionic conductivity of the CPE film. Hence, destruction of cathode is the principal reason leading to the failure of the SSLiB. The cathode binder can be replaced by PVDF to alleviate the oxidation induced failure of NBR and enhance the cell performance. Currently, optimization of the cell performance is still in our progress. The cycling performance of the pouch cell run under 60°C is presented in Figure 5F. After 10 cycles, the capacity remains at 38.4% of the first cycle, and this demonstrates that our technique is reliable for the fabrication of a pouch type SSLiB.

Currently, the critical current density of the LiRAP-NBR CPE system is 0.5 V, which can be further improved. That is because, on one hand, the room-temperature Li ion conductivity of the LiRAP-NBR CPE system is still not high enough. On the other hand, the mechanical strength of NBR still needs further improvement. Consideration of the practical application of the CPE film in SSLiB, the RT ionic conductivity of CPE film should be higher than 10^–3^ S cm^−1^. The CPE film should be mechanically robust to adapt to the process of SSLiB and tolerance to Li dendrites. Introduction of the conjugated polymer such as succinonitrile and polyvinyl chloride into NBR can be explored to enhance the Li ion conductivity and the mechanical strength of the CPE film. Surface coating of cathode with ionic conductor, e.g., LiNbO_3_, can be done to enhance the interface stability between cathode and NBR. Optimization of the electrochemical and mechanical properties of CPE films as well as the electrochemical performance of the SSLiBs are still in our progress.

## Conclusion

In summary, we have successfully developed a robust slurry-based procedure to prepare LOCB based flexible CPE film for sheet-type SSLiB. With low-polarity solvent, LOCB can be well stabilized during the slurry processing. As prepared LOCB/NBR CPE film exhibit good flexibility, improved electrochemical stability, enhanced thermal stability, and increased critical current compared with the NBR based SPE film. Sheet-type pouch cells with Li foil anode, LOCB/NBR CPE film, and LFP cathode have been successfully achieved. The LFP cathode exhibited a high capacity of 137 mAh/g. This work develops a scalable route for processing LiRAP based CPE film and opens the door for preparation of LiRAP based sheet-type SSLiB toward practical applications.

## Data Availability

The original contributions presented in the study are included in the article/Supplementary Material, further inquiries can be directed to the corresponding authors.
