# Supplementary material for: Li-Rich Antiperovskite/Nitrile Butadiene Rubber Composite Electrolyte for Sheet-Type Solid-State Lithium Metal Battery
Source: Front Chem. 2021 Nov 15;9:744417. doi: 10.3389/fchem.2021.744417 (PMC8634478; doi:10.3389/fchem.2021.744417)
Supplement: Supplementary file 1 [file Presentation1.pdf]

Supporting information

Li-rich antiperovskite/ nitrile butadiene rubber composite electrolyte for sheet-type solid-state lithium metal battery

Juncao Bian, Huimin Yuan, Muqing Li, Sifan Ling, Bei Deng, Wen Luo, Xuedan Chen, Lihong Yin, Shuai Li, Long Kong, Ruo Zhao, Haibin Lin, Wei Xia, Yusheng Zhao*, Zhouguang Lu**

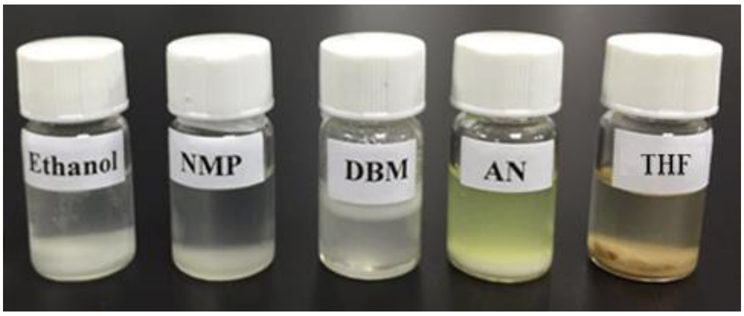

Figure S1 Photos of the LOCB stored in different organic solvents including N-methyl pyrrolidone (NMP), dibromomethane (DBM), acetonitrile (AN) and tetrahydrofuran (THF) for seven days.

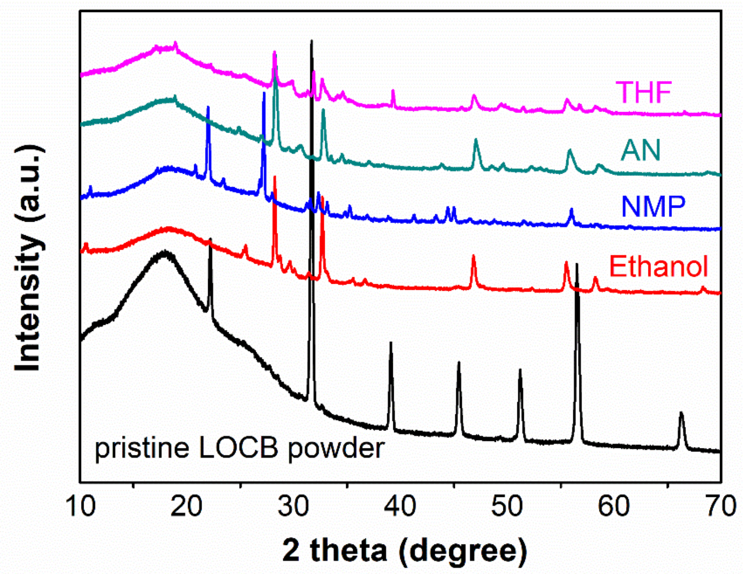

Figure S2 XRD patterns of the LOCB powder after stored in different organic solvent for seven days at room temperature.

The composition of the products are not investigated as they are beyond the scope of this work.

Table S1 The polarity of different organic solvents.

**Solvent**	ethanol	NMP	DBM	AN	THF	p-xylene
**Polarity**	4.3	6.7	3.8	6.2	4.2	2.5

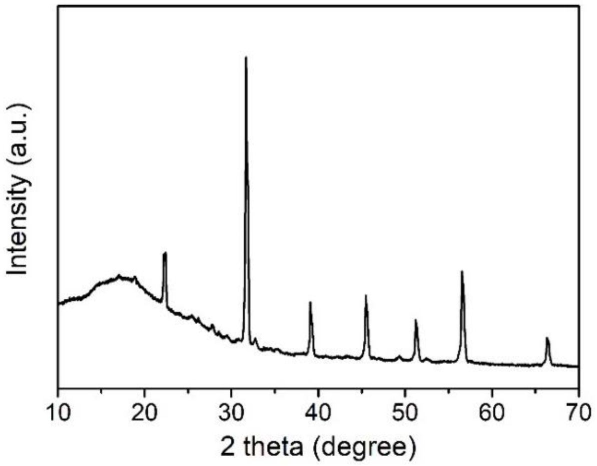

Figure S3 XRD pattern of LOCB after stored in xylene for seven days at room temperature.

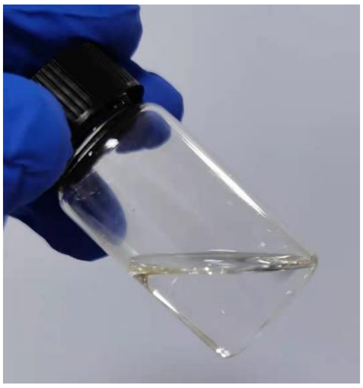

Figure S4 Photo of the mixture of DBM with TEGDME-LiTFSI solution.

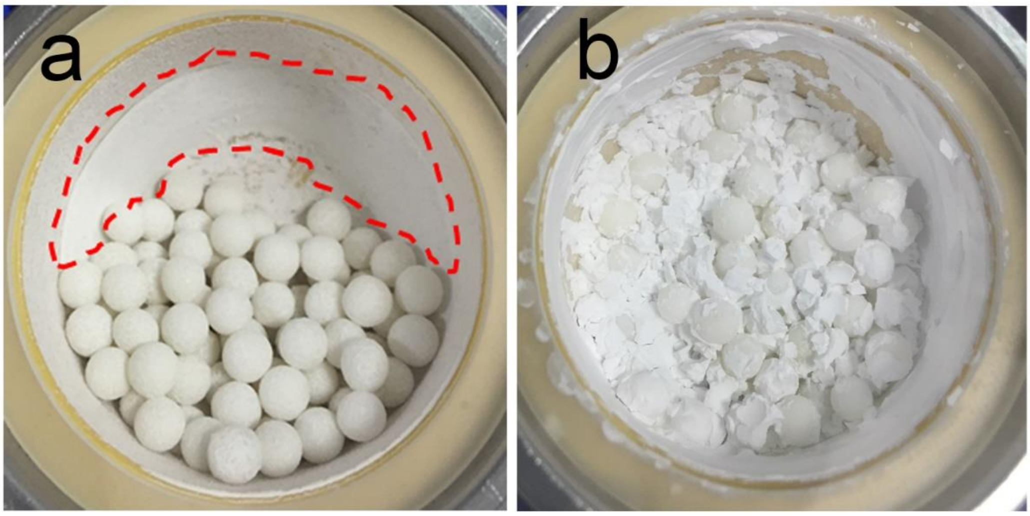

Figure S5 Photo of the milled LOCB. (a) Without adding DBM. (b) Adding DBM and dried.

LOCB powder tends to agglomerate during ball milling, which is highlighted by the red circle in Figure S5a. This problem can be addressed by adding some DBM during milling. During the milling process, on one hand, DBM plays the roles of dispersing agent and lubricant, which prevents

the agglomeration of the LOCB particles. On the other hand, the heat generated during the mechanical milling results in the evaporation of DBM, which provides an inert atmosphere protecting the LOCB powder from the contamination of the water molecules in the air. After vacuum drying, LOCB powders can be easily detached from the tank wall and milling balls, as shown in Figure S5b.

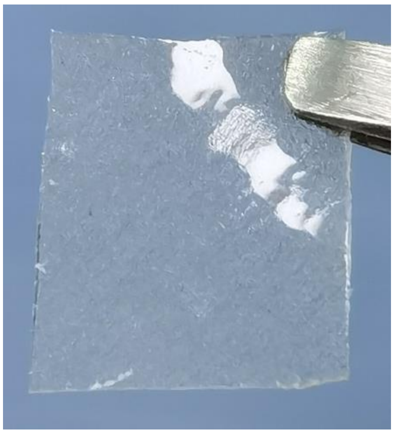

Figure S6. Photo of the pure NBR film.

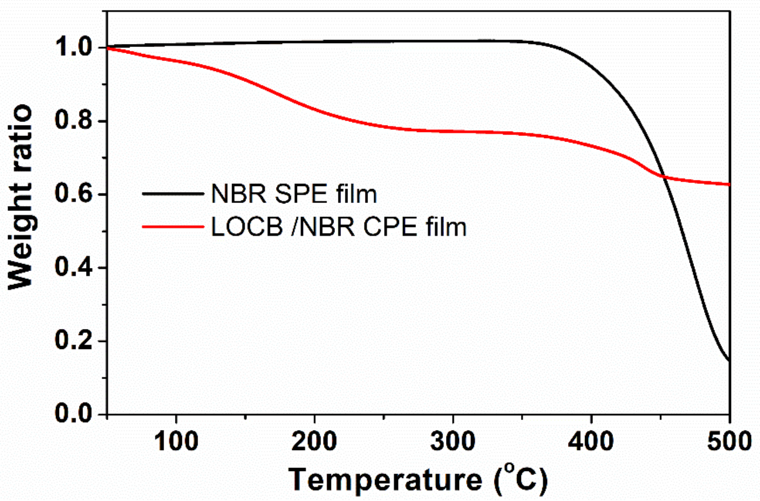

Figure S7. Thermogravimetric analysis curves of pure NBR film and LOCB /NBR CPE film.

The decrease in the weight of the CPE film before 200 °C is mainly due to the evaporation of the residual TEGDME in the film. When the temperature is above 356°C, NBR starts to decompose. The weight of it rapidly decreases to 15%. On the contrary, the weight is relatively stable after 450 °C for the 40% LOCB /NBR based CPE film. The weight remains 63% at 500 °C. It indicates that less vapor is generated under high temperature from CPE than that from NBR film, ensuring higher safety for the practical applications.

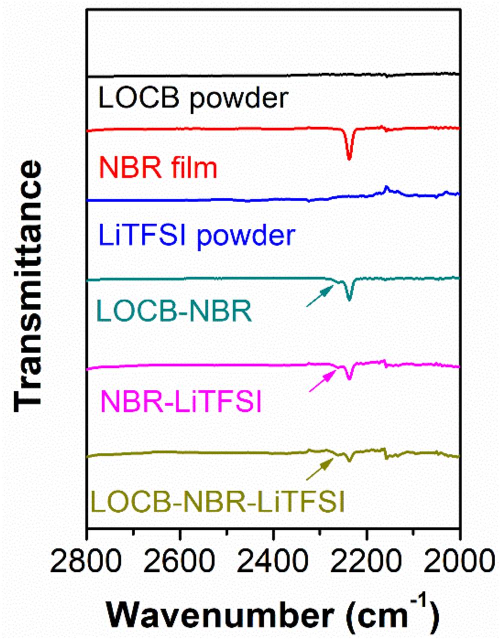

Figure S8 FTIR spectra of the different components of LOCB/ NBR CPE film and their mixtures in the range of 2800~2000 cm^{-1}. The arrows point to the peak at 2262 cm^{-1}.

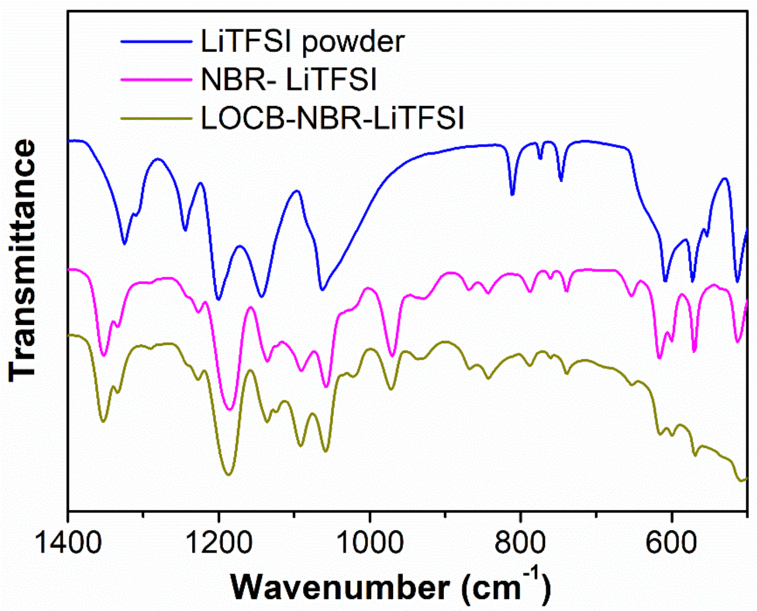

Figure S9 FTIR spectra of LiTFSI powder, NBR-LiTFSI mixture film and LOCB-NBR-LiTFSI film in the range of 1400~500 cm^{-1}.

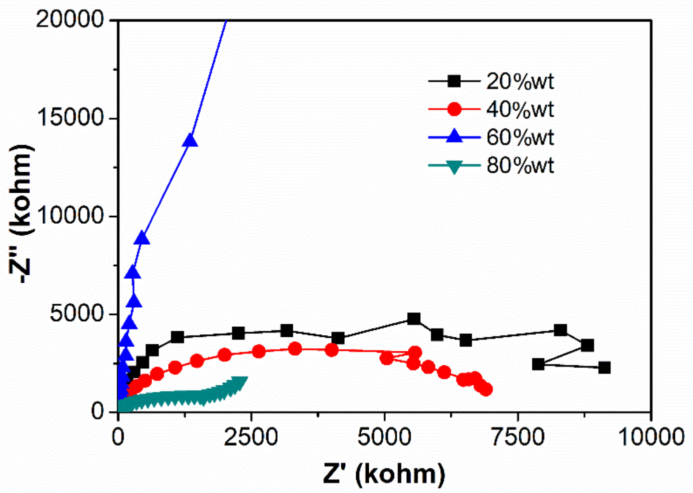

Figure S10 EIS plots of NBR-LOCB mixture films with different weight ratios of the LOCB measured at 60 °C. The ion conductivities at 60 °C are cal. 3.5×10^{-9}, 4.9×10^{-9}, and 1.8×10^{-9} 1.8×10^{-8} S cm^{-1} for 20wt.%, 40wt.%%, 60wt.% and 80wt.%, respectively.

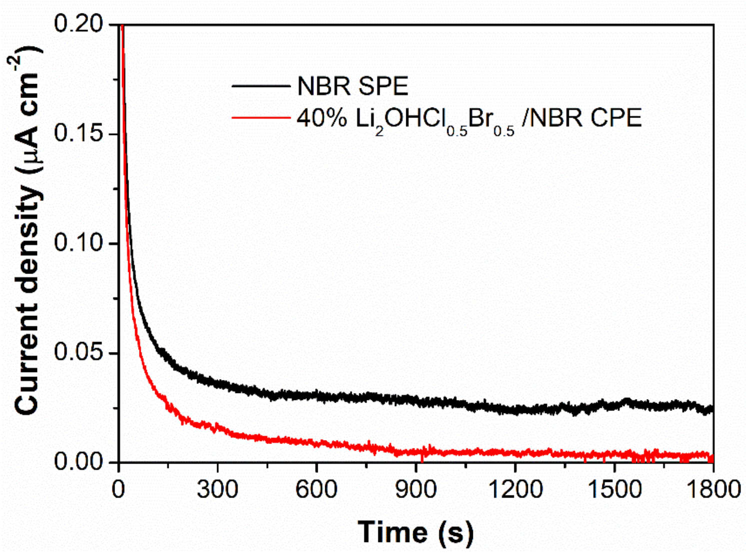

Figure S11 Polar curve of NBR SPE and 40% LOCB /NBR CPE film at 0.5 V for 1800 s.

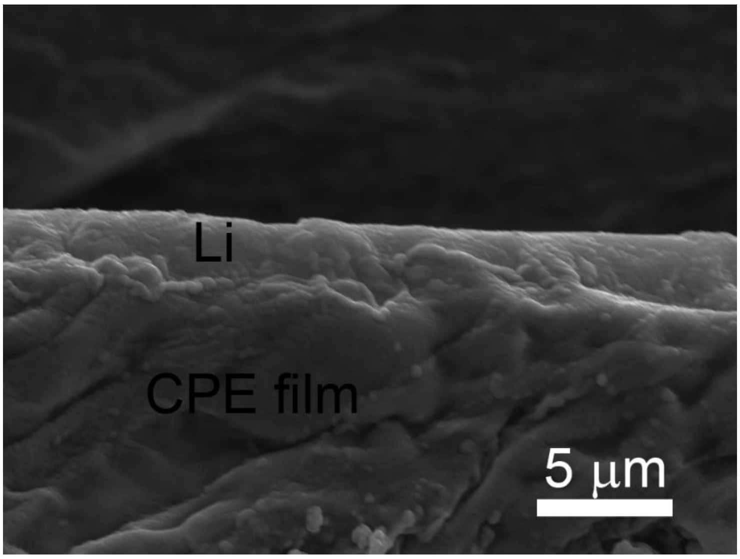

Figure S12 Cross-sectional morphology of Li and CPE film.

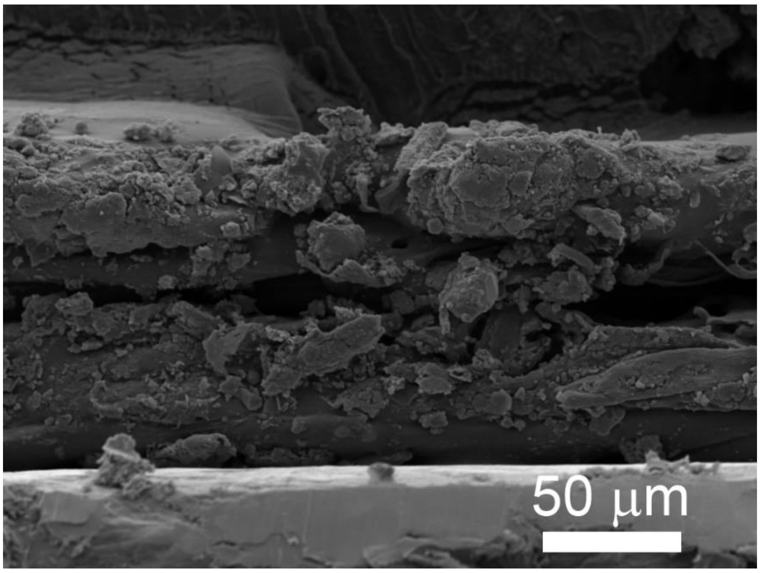

Figure S13 Cross-sectional morphology of cathode and CPE layers.

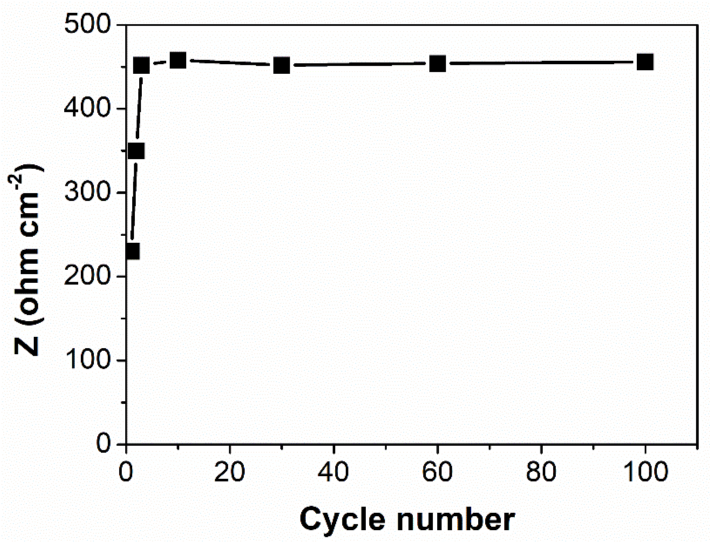

Figure S14 Interface resistance fitted from the EIS plots of the Li-Li symmetric cell after running for different cycles.
